# Interconnected exposure environments and potential TB transmission pathways among adolescents

**DOI:** 10.5588/ijtldopen.26.0373

**Published:** 2026-11-13

**Authors:** M. Amour, P. Joseph, P. Munseri, J. Ngowi, W. Akyoo, M.M. Amiri, C. Makunenge, E. Metta, C.R. Horsburgh, C. Whalen, E. Balandya, M. Matee, C. Fordham von Reyn

**Affiliations:** 1 Department of Community Health, School of Public Health and Social Sciences, Muhimbili University of Health, and Allied Sciences, Dar es Salaam, Tanzania;; 2 Department of Internal Medicine, School of Medicine, Muhimbili University of Health and Allied Sciences, Dar es Salaam, Tanzania;; 3 Department of Behavioural Sciences, School of Public Health and Social Sciences, Muhimbili University of Health and Allied Sciences, Dar es Salaam, Tanzania;; 4 Departments of Epidemiology, Biostatistics, Global Health and Medicine, Boston School of Public Health and Medicine, Boston, MA, USA;; 5 Global Health Institute, University of Georgia, Athens, GA, USA;; 6 Department of Physiology, School of Biomedical Sciences, Muhimbili University of Health and Allied Sciences, Dar es Salaam, Tanzania;; 7 Department of Microbiology and Immunology, School of Diagnostic Medicine, Muhimbili University of Health and Allied Sciences, Dar es Salaam, Tanzania;; 8 Infectious Disease and International Health, The Geisel School of Medicine at Dartmouth, Hanover, NH, USA.

**Keywords:** tuberculosis, adolescents, qualitative research, interferon-gamma release assay, community setting, Tanzania

## Abstract

**BACKGROUND:**

Adolescents represent an important target for TB control in high-burden urban settings, where infection may occur beyond the household. This study explored adolescents’ exposures across household, school, and community settings.

**METHODS:**

We conducted a qualitative sub-study nested within a prospective cohort to assess TB infection (TBI) among adolescents in Dar es Salaam. Serial interferon-gamma release assays classified participants into with prevalent, incident, or no TBI. Purposive sampling selected 21 adolescents, 13–17 years for in-depth interviews exploring TB-related exposure settings including household-built environments, school environment, commuting patterns, and social interactions. Interviews were conducted in Kiswahili, transcribed verbatim, translated, and analysed using thematic analysis.

**RESULTS:**

Our analysis revealed four interlinked exposure environments shaping TB risk. Households featured shared living and sleeping arrangements with limited ventilation facilitating sustained close-contact exposure. School environment involved sustained proximity among students in crowded classrooms. Daily commuting on public transport created recurrent high-contact exposure corridors. Community environments served as social mixing hubs, where respiratory symptoms were widely normalised.

**CONCLUSION:**

Adolescents in high-TB-burden settings encounter a broad range of contact environments as potential sources of TBI. These findings challenge traditional household-centric TB transmission models and underscore the need for multi-setting approaches to TB case-identification.

TB caused by *Mycobacterium tuberculosis* remains a major global public health challenge and continues to rank among the leading causes of death from a single infectious agent worldwide.^1^ In 2024, an estimated 10.7 million individuals developed TB, resulting in approximately 1.23 million deaths, and a global incidence of 131 per 100,000 population.^1^ Despite sustained global control efforts, transmission persists, largely driven by undiagnosed and untreated infection. To date, nearly two billion people worldwide are estimated to be infected, representing a substantial reservoir for future disease.^2^ Tanzania is classified as TB endemic and is among the 30 countries with the highest TB burden globally according to the World Health Organization (WHO).^3^ Although national data demonstrate progress, with TB incidence declining from 306 cases per 100,000 population in 2015 to 183 per 100,000 in 2023 and treatment success rates exceeding 96%, transmission remains ongoing.^4^ Within the country, Dar es Salaam consistently reports the highest TB notification rates, estimated at about 292 cases per 100,000 population, and contributed roughly to 15% of all notified TB cases in 2023.^5^

Although TB control strategies have traditionally focused on household transmission and adult disease, adolescents are increasingly recognised as a critical yet under-addressed population in the TB epidemic.^6^ Childhood bacille Calmette-Guérin immunisation provides protection against all forms of TB disease for more than 50 years in some settings,^7^ but efficacy wanes with time leaving many immunised adolescents susceptible.^8^ Adolescence represents a period of changing TB risk shaped by biological, behavioural, and social transitions. These include immunological and hormonal changes, expanding social networks, increased mobility, and prolonged exposure in congregate settings such as schools and community environments.^6,9,10^ TB risk during adolescence is also influenced by evolving patterns of exposure and transmission. Targeting adolescents through preventive services offers a potential triple dividend: reducing immediate morbidity, interrupting transmission during a highly socially connected life stage, and preventing progression to adult TB disease, thereby contributing to long-term epidemic control.^11^ Despite growing recognition that adolescents may serve as an important reservoir of recent TB infection (TBI) and contribute to community transmission, they remain largely excluded from routine TBI screening and preventive treatment strategies.^12,13^ Current national and the WHO guidelines primarily prioritise TB preventive therapy for defined high-risk groups, including young children and people living with HIV, with limited emphasis on systematic screening or prevention among adolescents.^14^ Urban environments pose additional challenges for TB control.^15^ Evidence from Tanzania and other high-burden settings suggests that adolescents frequently acquire TBI outside the household, including in schools, neighbourhoods, and informal social settings.^16,17^ These findings challenge the traditional focus on household contact tracing and highlight the need to better understand transmission pathways beyond domestic environments.^18^

This study, nested within an ongoing prospective cohort investigating TBI using T-SPOT.TB among school-going adolescents in Dar es Salaam, Tanzania, aims to explore potential pathways of TB transmission by examining household environments, school-related exposures, social interactions, and community settings.^19^ By characterising the everyday contexts in which adolescents interact and spend time, this study aims to generate evidence to inform effective adolescent-focused TB prevention and control strategies in high-burden urban settings.

## METHODS

This qualitative case study was nested within an ongoing 5-year prospective cohort study examining TBI among adolescents in Dar es Salaam, Tanzania (March 2023 to March 2028). Dar es Salaam is Tanzania’s largest city, with a population of approximately 5.4 million according to the 2022 National Census and is entirely urban in character. Administratively, it is divided into five districts; Ilala, Temeke, Kinondoni, Ubungo, and Kigamboni, and serves as the country’s economic and commercial capital. The region contributed approximately 17% of all TB cases notified nationally in 2024, with Temeke municipality alone contributing for nearly a third of all notifications within the city.^20^

The parent cohort is being conducted in schools across two high-burden districts, Temeke and Ilala, and aims to evaluate a novel case-finding approach by serially screening adolescents for TBI using T-SPOT.TB Interferon-Gamma Release Assays (IGRAs), followed by investigations of members of household of adolescents found with evidence of new TBI to identify potential sources of active TB disease.

The qualitative study explored adolescents’ lived experiences and social contexts of potential TB exposure across three IGRA-defined infection groups: 1) prevalent TBI, defined as IGRA-positive at enrolment; 2) incident TBI, defined as conversion from IGRA-negative to IGRA-positive during follow-up; and 3) persistently negative TBI, defined as remaining IGRA-negative throughout follow-up and serving as the comparison group. The participants had between 4 and 12 months of follow-up before the qualitative study. Adolescents were defined as secondary school students aged 13–17 years.

### Sampling

We used purposive heterogeneous (maximum variation) sampling to recruit adolescents from the three IGRA-defined groups: prevalent TBI, incident TBI, and persistently negative TBI, to capture diverse experiences across infection status and social contexts.^21^ A total of 21 adolescents were selected. The selection ensured variation in age, sex, residential setting, and reported history of household or close contact with a person diagnosed with active TB. Sampling was conducted iteratively alongside data collection and analysis. After each round of interviews, the research team reviewed emerging codes and themes to assess whether new insights relevant to the study objectives were identified. Data collection ceased when successive interviews yielded no new themes or meaningful variation in the identified exposure settings and continued until thematic saturation was reached.^22,23^

### Selection and recruitment strategies

Participants were recruited following receipt of T-SPOT.TB IGRA results during routine school-based cohort visits. Eligible adolescents were approached at their respective schools and invited to participate in in-depth interviews. Prior to recruitment, the research team met with school administrators to explain study procedures and ensure that participation did not interfere with academic activities.

### Data collection

Interviews were conducted between March 2025 and May 2025 in private, pre-identified locations within school premises to ensure confidentiality and minimise interruptions. They were conducted in Kiswahili by a multidisciplinary team comprising of a study nurse and medical doctors (AA, PJ, MMA), and experienced qualitative research assistants (AM, WA). Interviews lasted approximately 30–50 min, were audio-recorded, and were contextualised using demographic data collected during cohort enrolment. Audio recordings were transcribed verbatim, translated to English and verified against the original audio before analysis. A semi-structured interview guide was developed by adapting domains from a previously validated quantitative TB risk-factor questionnaire into open-ended qualitative questions aligned with study objectives. Domains explored included household and living conditions, TB exposure history, neighbourhood environments, daily mobility patterns, and social contexts shaping adolescents’ interactions. Flexible probing was used to elicit detailed context-rich accounts.

### Data analysis

Qualitative data were analysed using a thematic analysis as described by Braun and Clarke (2006).^24^ Given the objective of identifying settings in which adolescents may be exposed to TB, analysis was primarily deductive. Coding was initially guided by research questions, while remaining open to unexpected patterns emerging from the data. Additionally, transcripts were read repeatedly to achieve familiarisation with the data before initial coding. Codes were compared across transcripts and iteratively refined through discussion within the research team. A preliminary codebook was developed and independently reviewed by PJ and AM with discrepancies discussed and resolved through consensus. Following codebook finalisation, all transcripts were systematically coded using Microsoft Excel. Codes were subsequently organised into themes and representative quotations were selected to illustrate key findings while preserving participants’ perspectives.

### Ethical statement

Ethical approval was obtained from the Tanzania National Institute for Medical Research (NIMR/HQ/R.8a/Vol.IX/4807), the Muhimbili University of Health and Allied Sciences Research Ethics Committee (MUHAS-REC-10-1427), and the Dartmouth Committee for the Protection of Human Subjects. Additional permissions were granted by the Tanzania President’s Office of Regional Administration and Local Government and the Ministry of Education, Science and Technology. Written parental consent and adolescent assent were obtained before the interviews. Participants received age-appropriate information about the study, including objectives, procedures, confidentiality, and their rights, and were informed that participation was voluntary and withdrawal was permitted at any time.^25^ Confidentiality was maintained through identification numbers and secure handling of study data accessible only to the research team.

## RESULTS

This study included 21 adolescent secondary school students from Dar es Salaam, all of whom were day scholars. Participants were aged 14–17 years, with the majority (47.6%) aged 15 years. Gender distribution was nearly even, with 11 adolescent boys and 10 adolescent girls. Most participants were in the second year (form two; 61.9%) and the rest were in the third year (form three) of the 4-year secondary education. Based on IGRA classification, 14 participants had incident TBI acquired during follow-up, 5 had prevalent TBI at enrolment, and 2 had no TBI/persistent negative IGRA. More than half of participants resided in Temeke district (52.4%), followed by Ilala (33.3%), and a smaller number from Ubungo, Kigamboni, and Kinondoni districts (each 4.7%; Table 1).

**Table 1.** Characteristics of study participants.

Variable	n = 21 (%)
Age (years)	
14	7 (33.3%)
15	10 (47.6%)
16	3 (14.3%)
17	1 (4.7%)
Gender	
Adolescent boy	11 (52.4%)
Adolescent girl	10 (47.6%)
Class	
Form two	13 (61.9%)
Form three	8 (38.1%)
TB infection category based on IGRA	
Incident TB infection	14 (66.7%)
Prevalent TB infection	5 (23.8%)
No TB infection	2 (9.5%)
Residence (district)	
Temeke	11 (52.4%)
Ilala	7 (33.3%)
Ubungo	1 (4.7%)
Kigamboni	1 (4.7%)
Kinondoni	1 (4.7%)

IGRA = interferon-gamma release assay.

Participants described daily routines that spanned interconnected environments, including households, schools, public transport, and community spaces. Across these spaces, participants described shared indoor living and sleeping arrangements at home, sustained close contact during school routines, and regular movement through crowded transport systems. These experiences extended into community settings, where both brief and prolonged social interactions were common across a range of indoor and outdoor environments.

### Shared indoor living and sleeping arrangements

Participants described household environments characterised by shared indoor living and sleeping arrangements, often involving multiple family members within limited space. Most participants lived with parents and siblings, while others described extended family settings that included grandparents, aunts, uncles, or other relatives. Across these arrangements, sharing of rooms was common, with several adolescents reporting that more than four individuals were occupying a single room. In some cases, sleeping spaces were further constrained, with multiple people sharing a bed or using mattresses on the floor, as one responded reported:

I share a room with my two brothers, two uncles, and another uncle who does not sleep there often … We are about six. Four sleeps in one bed, and the others sleep on a mattress spread on the floor.

A 15-year-old girl, prevalent TBI

Participants also described variation in ventilation within these shared spaces. Many reported having one or two windows, which were typically opened during the day and closed at night. Some noted limitations such as blocked or poorly functioning windows, resulting in reduced airflow. A few adolescents described relying on fans, while others reported that rooms could feel ‘heavy’ or ‘stuffy’, particularly when crowded or filled with household items.

The window is there but not properly fixed. They blocked it with iron sheets. It hasn’t been repaired properly, so only faint light and air enter unless you open the door, so I sometimes use a fan.

A 15-year-old girl, prevalent TBI

In addition, several adolescents reported living with or having previously lived with someone diagnosed with TB, either within their household or during visits to relatives. A small number described close and prolonged interactions during such periods, including sharing a bed.

### Sustained close contact in school routines

Beyond the household, adolescents described spending a substantial portion of their day in school environments, where proximity to others was consistent. Participants reported large class sizes, typically ranging from 50 to 80 students, in classrooms designed to accommodate up to 45 individuals. Seating arrangements often involved three to four students sharing a desk, resulting in limited personal space during lessons, and some reported having occasional combined classes for some subjects, which make classrooms even denser. In some cases, increasing class sizes led to relocation to larger rooms, although adolescents noted that space constraints persisted despite these adjustments.

Right now, there are many of us; this is the year with the most students, our year. Science is the class with the highest number of students. Here, we are 82. We were moved because these rooms are slightly wider.

A 15-year-old girl, incident TBI

These dense seating arrangements made coughing a common occurrence during school activities. One participant noted:

Coughing is normal because we stay close together with little space, so it’s just something natural.

A 16-year-old boy, incident TBI

In addition to regular classroom activities, adolescents described participation in other school-based events that brought together larger groups of students. These included assemblies, debates, religious sessions, and club meetings, often involving mixing of students across classes or grades. Some activities involved very large gatherings, with participants estimating attendance in the hundreds, often in open spaces such as assembly grounds as described by one participant:

There are many people because it involves the whole school … even five hundred students can attend. We do this every Friday.

A 15-year-old boy, prevalent TBI

### Daily movement through crowded transport systems

Movement between home and school further extended adolescents’ daily interactions into public transport systems. Participants consistently described relying on minibuses (*daladala*) and trains as their main means of commuting. These journeys were described as routine, typically occurring twice daily. Transport environments were characterised by crowding and limited space, often making it difficult to avoid close physical contact with others. Adolescents described vehicles as frequently filled beyond seating capacity, and passengers stand in proximity.

In the daladala (minibus), it is a problem because they load too many passengers, and it becomes hard to breathe. Some people even block the windows.

A 15-year-old boy, prevalent TBI

In addition, train travel was described as particularly congested, especially near major stops and rush hours, where large numbers of passengers boarded within a short time. Ventilation conditions were also described as variable, with some adolescents noting that windows were small, blocked, or kept closed, contributing to a warm and enclosed environment. They noted that movement within the carriage was often restricted due to crowding.

The train has huge crowds … you do not get a seat … you are stuck. The crowd is worse than in the bus.

A 16-year-old boy, incident TBI

These commuting experiences were described as a regular extension of daily routines, linking household and school environments through repeated exposure to crowded shared spaces.

### Extended social interaction in community spaces

Outside of school hours, participants described engaging in a range of community and social activities that formed part of their daily or weekly routines. These included visiting markets, neighbourhood shops, food vending areas, religious gatherings, and informal entertainment spaces such as movie-loading libraries and local viewing halls. These environments varied in size, ventilation, and duration of stay but commonly involved close contact with other people. Interactions within these settings varied in duration. Some, such as visits to markets or shops, were described as brief but frequent, often involving movement through crowded spaces. One participant noted:

At the market, there is a lot of crowding. The crowd is big, many people.

A 15-year-old girl, incident TBI

Other environments involved more prolonged stays. Small indoor spaces such as movie-loading libraries were described as enclosed and frequently crowded, sometimes influencing how long adolescents remained in or whether they chose to return later when it is less crowded.

Near home, just a short distance, there is a library … There are many people, so many that sometimes we get fed up and don’t load movies.

A 15-year-old boy, prevalent TBI

Other forms of entertainment were also described, including visits to informal movie-viewing spaces locally known as *kibandaumiza*. These were characterised as small indoor settings that often attracted tightly packed groups, mainly young adults, where individuals remained for extended periods.

Additionally, some participants described participating in outdoor social activities such as informal football games near home, which typically involved smaller groups and open spaces. A few participants also reported attending football matches at large stadiums, where they described very high attendance and dense crowds. One described their experience during a local match at the national stadium:

Some days when Yanga plays, my father and I go to the stadium to watch … The last time was at the Benjamin Mkapa Stadium. The crowd is always huge. On Yanga Day, the crowd is always big.

A 15-year-old boy, incident TBI

Similarly, religious activities, including attendance at church, mosque, or madrasa, were described as regular and structured gatherings, sometimes involving large groups seated together in indoor or semi-enclosed spaces.

At the madrasa, there are so many of us, I cannot count them; they are many. The place is big, and we all sit together in a large group.

A 14-year-old girl, incident TBI

In some instances, participants described encountering individuals with visible symptoms such as cough in public places such as shops and markets and in public transport. These interactions were generally perceived as a normal part of everyday activities, particularly when they were brief.

Hearing someone cough is normal, but you do not know if they cough every day … I was buying something and finished, then someone came, and I saw and heard him coughing heavily.

A 16-year-old boy, incident TBI

## DISCUSSION

This study explored the spectrum of adolescent exposures that may contribute to TBI in an urban, high-burden setting. The findings demonstrate that adolescent exposure to TBI is not confined to the household but occurs across multiple interconnected environments, including schools, public transport systems, and community spaces. These settings are linked through adolescents’ routine daily movements suggesting that exposures may accumulate across environments rather than arising from a single location.

Our findings indicate that the household environment remains an important source of TB, among adolescents, particularly in contexts characterised by shared sleeping arrangements, prolonged indoor contact, and limited ventilation. Consistent with prior evidence, one study identified the household as a potential source of infection for adolescents aged 10–14 years, highlighting the importance of household exposure in this age group.^11^ However, household exposure alone does not fully account for transmission dynamics as a substantial proportion of exposed individuals do not become infected, suggesting outside sources as primary drivers of transmission.^18,26,27^ Our study findings extend this interpretation by demonstrating that household exposure forms part of a broader, continuous exposure continuum shaped by adolescents’ daily mobility across environments. These findings reflect that household exposure forms part of a broader, continuous exposure continuum shaped by adolescents’ daily mobility across environments. These diverse social environments characterise adolescents, although similar exposure opportunities may also occur among other urban residents.^28^

School environments emerged as an important setting where adolescents reported spending substantial time in close contact with peers. High student density, prolonged classroom time, and routine mixing during school activities create sustained conditions for close contact. This aligns with evidence from China and Serbia identifying schools as congregate settings where shared space increased the risk for TB transmission.^29–31^ Notably, participants in this study described coughing as a common, unremarkable occurrence within classrooms, a normalisation of respiratory symptoms that may blunt risk perception and sustain high-contact interactions in already dense environments.

Community settings add further complexity with adolescents engaging in a range of social activities that involve both brief and prolonged interactions. Informal indoor settings, such as small recreational venues and gathering spaces, were described as environments where close contact occurs beyond immediate social networks. Adolescents also described encountering individuals with cough symptoms in public spaces, often perceiving these interactions as a normal part of everyday life. Collectively, these observations suggest that community settings function as repeated social mixing hubs where exposure opportunities are both frequent and socially normalised. These observations align with evidence from Tanzania linking informal social interactions to increased TBI risk among adolescents and young adults.^17^

Public transport represents another high-risk corridor. Overcrowding characterised by poorly ventilated buses creates conditions of close, often unavoidable proximity to potentially infectious individuals particularly relevant for adolescents who commute daily to school especially when passengers are symptomatic and coughing. Evidence from a systematic review of contact investigations demonstrated that daily commuting in poorly ventilated public transport settings can facilitate TB transmission.^32,33^ In this context, transport systems function as high-frequency exposure corridors that connect households, schools, and community environments into a continuous transmission network.

Beyond these identifiable settings, adolescents may also encounter TB through transient casual contact with infectious individual’s unknown to them. Unlike household, close-contact, or community transmission, which is more readily recalled and reported, these transient interactions occurring in shared and crowded places are by nature invisible to those exposed.^34^ Since adolescence and early adulthood are periods of heightened TBI risk, they may be exposed repeatedly without awareness, through brief or circumstantial proximity to undiagnosed or untreated cases in their daily environments.^34,35^ This form of exposure is difficult to capture through self-report methods. Community network studies in high-burden urban African settings have further demonstrated that transmission chains frequently extend beyond household and social contacts into broader community networks.^35^

Several limitations should be considered. This research was not designed to quantify transmission risk or establish direction of infection, limiting direct comparison with epidemiological estimates. Findings relied on participant self-report and recall, which may be subjected to reporting bias. Identified locations reflect the settings that adolescents were willing and able to recall and disclose, and this should be interpreted as the perceived exposure environments visited by participants. Adolescents may have underreported certain settings or activities perceived as socially undesirable, leaving the full spectrum of exposure environments unknown. Furthermore, the study was not designed to capture incidental contact with infectious individual’s unknown to the participant. The study was conducted among day-school adolescents in an urban setting, and therefore findings may not be transferable to out-of-school youth, boarding students, or adolescents living in different geographic contexts within Tanzania. The study also does not directly measure microbiological transmission linkage between settings.

## CONCLUSION

Taken together, these findings support a network-based understanding of TB exposure among adolescents, where risk is shaped by repeated interactions across multiple interconnected social environments rather than a single source.^36^ This challenges household-centric prevention models and support multi-setting strategies that extend beyond the home. Specifically, this could include school-based health education, mobility-informed contact investigation, improved ventilation in congregate settings, and community awareness campaigns around persistent cough, collectively strengthening adolescent-responsive TB services. Future research should quantify the relative contribution of different exposure settings and evaluate interventions targeting the social environments in which adolescents spend their daily lives.
